# Flight energetics of barn swallows across speeds: high aerodynamic efficiency, but low energy conversion efficiency

**DOI:** 10.1242/jeb.252390

**Published:** 2026-09-17

**Authors:** Pablo Macías-Torres, Sonja I. Friman, L. Christoffer Johansson, Anders Hedenström

**Affiliations:** Department of Biology, Lund University, 223 62 Lund, Sweden

**Keywords:** Bird flight, Flapping flight, *Hirundo rustica*, Metabolic power, Mechanical power, Wind tunnel

## Abstract

Bird flight conversion efficiency (η) determines the amount of metabolic power (*P*_met_) transformed into mechanical power (*P*_mech_) to remain aloft, and it is central to understanding animal flight energetics, yet it remains difficult to quantify. Using ^13^C-labelled sodium bicarbonate (NaBi) and particle image velocimetry (PIV), we measured *P*_met_ and *P*_mech_, respectively, in un-instrumented barn swallows (*Hirundo rustica*) flying across 7.8 to 13.6 m s^−1^ in a wind tunnel. Both *P*_met_ and *P*_mech_ showed the expected U-shaped relationship with airspeed. Whole-animal η showed a shallow peak at 7% at 11 m s^−1^. Partial efficiency (η_p_), estimated from horizontal and 4 deg climbing flight, was 27% (95% confidence interval CI: −2 to 56.1). Compared with thrush nightingales previously studied under identical conditions, barn swallows exhibited higher weight-specific flight *P*_met_ but lower lift-specific *P*_mech_, reflecting a higher basal metabolic rate but greater aerodynamic efficiency in barn swallows. Barn swallows showed lower peak and flatter η curve across speeds compared with that of the nightingales, suggesting a trade-off between flight energetics and flight speed: higher efficiency peak over a narrow speed range (thrush nightingales) versus lower, more consistent efficiency across a broader speed range (barn swallows). Whereas nightingales fly mainly during migration, barn swallows spend much of the day airborne and use a wider range of flight speeds, potentially favouring versatility over a narrow η peak. Although η has been studied in few species, our results suggest that η is both species and speed dependent, probably shaped by species-specific ecological demands.

## Teaser

**Summary:** Barn swallows combine a high aerodynamic efficiency with a high flight metabolic power. This results in a relatively low energy conversion efficiency compared with other species studied under the same circumstances. Flight energetics seem to be shaped by species-specific ecology and flight behaviour.

## INTRODUCTION

Flight is both an effective (high-speed movement) and efficient (low cost of transport) mode of locomotion, but quantifying the energy required to fly in animals is a challenge (Bishop and Guglielmo, 2022; Schmidt-Nielsen, 1972). Flying animals sustain flight by flapping their wings to accelerate the surrounding air, generating both lift and thrust, while adding kinetic energy to the air. This is fuelled by metabolic energy input, which includes the energy expended by the flight muscles and other body systems involved in sustaining flight. The ratio between these two quantities defines the energy conversion efficiency of flight (η), and quantifies how much chemical energy is shunted into mechanical work, needed to remain aloft, by the flying animal (Hedenström, 2025).

Until recently, flight energy conversion efficiency, a critical component in understanding the energetics of flight, has not been possible to quantify directly (but see Currie et al., 2023; Hedh et al., 2020; Macías-Torres et al., 2025). This is mainly due to the technical challenges of accurately capturing both metabolic and mechanical power (*P*_met_ and *P*_mech_, respectively) in freely flying animals (Hedenström and Lindström, 2017). Traditional methods often relied on indirect estimates or flight models with simplified aerodynamic approximations (e.g. Pennycuick, 2008). Recent methodological advances have allowed for measuring both *P*_met_ (Hambly et al., 2002) and *P*_mech_ (von Busse et al., 2014), in un-instrumented flying animals under near-natural flight conditions in the same individuals in wind tunnels.

In this study, we quantified whole-body flight conversion efficiency in barn swallows (*Hirundo rustica*) using *in vivo* measurements of both *P*_met_ and *P*_mech_. *P*_met_ was assessed via the ^13^C-labelled sodium bicarbonate (NaBi) method, while *P*_mech_ was calculated as aerodynamic power using particle image velocimetry (PIV) which captures the bird's wake during flight. Additionally, we estimated partial efficiency (η_p_), by having the birds fly horizontally and at a climbing angle through tilting the wind tunnel. This approach was used to derive the first-ever estimate for flight efficiency, which has remained in use until now and is based on conversion efficiency being related to the relative change in *P*_met_ and *P*_mech_ required for a known climbing flight angle (Bernstein et al., 1973; Tucker, 1972; Pennycuick, 2008).

Barn swallows are highly aerial birds, spending much of their daily activity in flight while foraging, commuting and migrating (Turner, 2006). Barn swallows differ from other passerines by their wing morphology, having relatively long, pointed and comparatively high aspect ratio wings, a wing index associated with reduced induced drag and high lift production (Bruderer et al., 2001). Their wing design allows barn swallows to use gliding phases effectively between active flapping bouts, contributing to their characteristic intermittent flight style, compared with continuous or flap-bounding flapping flight in most other passerines (Bruderer et al., 2010, 2001; Tobalske, 2010). Given the barn swallow's morphological and behavioural adaptations for aerial life (Turner, 2006), we hypothesised that this species will exhibit a relatively high energy conversion efficiency. We further predicted that, consistent with prior flight energy conversion efficiency measurements in migratory songbirds, to achieve low costs of transportation and migration (Macías-Torres et al., 2025), energy conversion efficiency in barn swallows will show a peak at intermediate flight speeds, reflecting optimisation for ecologically relevant speeds.

**Table:** 

**List of symbols and abbreviations**	
BMR	basal metabolic rate
COT	cost of transport
*F*_eff_	effective wingbeat frequency
***g***	gravitational acceleration
*k*_c_	isotope elimination rate
*m*	body mass
NaBi	^13^C-labelled sodium bicarbonate
*P*_mech_	mechanical flight power
*P*_mech_^*^	lift-specific mechanical flight power
*P*_met_	metabolic flight power
*P*_met_^*^	weight-specific metabolic flight power
*P*_mp_	flight power at minimum power speed
*P*_rec_	post-flight recovery metabolic power
PIV	particle image velocimetry
RMR	resting metabolic rate
*U*	airspeed
*U*_mp_	minimum power speed
*U*_η,max_	airspeed at maximum whole-animal energy conversion efficiency
η	whole-animal energy conversion efficiency
η_fm_	flight muscle conversion efficiency
η_fm,recovery_	*P*_rec_ based flight muscle converison efficiency
η_max_	maximum whole-animal energy conversion efficiency
η_p_	partial energy conversion efficiency
θ	wind-tunnel inclination angle

## MATERIALS AND METHODS

We captured 12 barn swallows, *Hirundo rustica* Linnaeus 1758, at lake Krankesjön, South Sweden (55°42′N, 13°27′E), on 4 September 2023. Six birds, all juveniles, adapted to the conditions in captivity and successfully progressed in their flight training in the wind tunnel, while the other six birds were released. The birds were kept in a communal aviary, with a day:night light routine of 13.5 h light:10.5 h dark, simulating local daytime conditions at the time of capture. Water and food were provided *ad libitum* in a half-cylinder plastic feeder suspended from the ceiling, and consisted of live mealworms (*Tenebrio molitor*) and frozen buffalo worms (*Alphitobius diaperinus*). Mean body mass upon capture was 21 g (range: 18.7 to 23.6 g). During the experiments, the average body mass was 17.9 g (range: 15.7 to 20.7 g).

Flight power measurements were obtained for flight speeds ranging from 7.9 to 13.6 m s^−1^ for *P*_met_ and between 7.8 and 13.1 m s^−1^ for *P*_mech_. All flight experiments were performed during the daytime. We used dimmed light conditions inside the wind tunnel to minimise the birds' stress, preventing them from seeing the flight operators and to facilitate their recapture after each flight experiment. Additionally, a thin plastic net (mesh approximately 15×15 mm; 0.1 mm chord thickness) was placed upstream to prevent birds from entering the settling chamber, where speed is reduced in the contraction section in front of the wind tunnel's testing section (Pennycuick et al., 1997). Barn swallows were removed from the cage in the morning and fasted for at least 4 h before a flight experiment to ensure post-absorption state and promote fat-oxidising metabolism (Jenni-Eiermann and Jenni, 2012). All birds were kept together in a dark cage during the fasting period, and each bird was returned to the aviary after its flight experiment. Prior to each experiment, the birds were weighed (±0.01 g). After the completion of all the experiments, the birds were released back into the wild and flew away seemingly unaffected. Permission for the experiments was approved by the Malmö-Lund Animal Experimentation Ethics Board (5.8.1817907/2023).

### Measuring flight *P*_mech_ – PIV

To visualise the flow behind the bird, we used a stereo-PIV setup similar to that in Johansson et al. (2018) with four high-speed cameras placed downstream of the intended flight location of the bird (Nova R2, Photron Deutschland GmbH, Reutlingen, Germany) and a pulsed laser generating a light sheet (LDY304PIV laser, Litron Lasers Ltd, Rugby, UK) oriented perpendicularly to the wind direction, also downstream of the intended flight location. The dual pulsed laser was operated at 720 Hz and the camera array recorded the illuminated aerosol particles (di-ethyl-hexyl-sebacate, 1 μm diameter) that were previously seeded into the wind tunnel air. Processing of the recordings and vector extraction were done using DaVis (v.10.2.1, LaVision GmbH, Gottingen, Germany). Only three of the four cameras could be used for the processing because one of the cameras had been disturbed during the experiments.

The stereo-PIV processing was performed, as described in the following steps, using the GPU option. The velocity vectors were calculated with a window shift in the stream-wise direction of 4 pixels for the initial pass, using an initial box size of 96×96 with two passes, followed by a final box size of 32×32 with three passes, with an overlap of 50%. Between passes, a twice-applied universal outlier detection was used: vectors that deviated more than 2 standard deviations (s.d.) from their neighbours in a filter region of 5×5 were replaced by the next-best vectors if those deviated by less than 3 s.d. The vectors were filtered by correlation value >0.25, and a vector validation was applied where vectors were accepted if the stereo reconstruction error was below 1 pixel. Finally, anisotropic denoising was applied, with the setting ‘weak’. The final vector volume size was 322×343 mm with a vector spacing of 2.4 mm (∼4.2 vectors cm^−1^), thus yielding 135×140 vectors.

*P*_mech_ was defined as the rate at which kinetic energy was added to the wake for an integer number of wingbeats, assuming inertial power to be insignificant (Pennycuick, 2008; Pennycuick et al., 2000). *P*_mech_ was estimated from the PIV-generated velocity fields of the bird wakes, following the description in Johansson et al. (2018) and Macías-Torres et al. (2025), with the following changes. We used the ‘automask’ described in Johansson and Henningsson (2021) to isolate the wake vorticity, which was then used to reconstruct the velocity field beyond the wind tunnel limits to a square size of 2.4×2.4 m using the Helmholtz–Hedge decomposition (von Busse et al., 2014). The original measurements inside the mask were reinserted into the reconstructed flow field before calculating the kinetic energy in the flow. This method effectively removes noisy background flow while maintaining the vorticity associated with the animal wake. In addition to *P*_mech_, vertical (lift) and horizontal (thrust) forces were estimated using the masked vorticity as applied in Johansson (2024). Calculations were performed using custom written code in MATLAB 2024b (MathWorks Inc.).

In barn swallows, the flight kinematics deviate from continuous flapping and often include brief upstroke interruptions between wingbeats (Bruderer et al., 2001; Park et al., 2001), which may lead to increased forces and power output during the active beats to compensate for these pauses. Unlike previous studies from our lab (Hedh et al., 2020; Macías-Torres et al., 2025), we did not apply a filtering step to exclude PIV measurements where the vertical force deviated more than ±20% from the bird's weight. This decision was based on the fact that our analysis focused solely on active wingbeat sequences, as the pauses in between wingbeats were rarely recorded in our PIV set up. Had we applied the filter, six measurements would have been excluded, indicating that our estimates of *P*_mech_, if anything, are probably biased upward rather than being underestimated.

To contextualise the limitation of estimating *P*_mech_ from active wingbeats only, we report the wingbeat frequency across airspeeds in the sequences used for our *P*_mech_ analysis. We then compared these values with the effective wingbeat frequencies reported by Bruderer et al. (2001) for barn swallows flying horizontally in a different wind tunnel. In contrast to our measurements, Bruderer et al. (2001) estimated wingbeat frequency over 20 s intervals, thereby incorporating both active flapping phases and pauses between wingbeats.

### Measuring flight *P*_met_ – NaBi

*P*_met_ was measured using the ^13^C-NaBi technique, following the same protocol described in Macías-Torres et al. (2025). This method uses the elimination rate of previously injected isotopic carbon as a proxy for CO_2_ production when direct respirometry measurement of CO_2_ is not available, such as when flying freely in a wind tunnel. First, an isotopic carbon solution (0.56 mol l^−1^ NaH_2_^13^CO_3_; Sigma-Aldrich) was injected subcutaneously into the bird. The bird was then transferred to a darkened respirometry chamber of 1.5 l volume through which CO_2_-free air (21% O_2_, balance N_2_) from a gas cylinder (Linde Gas AB, Solna, Sweden) passed at a constant rate of 2 l min^−1^. The airstream was subsampled by an isotope analyser (PICARRO – G2201-I, Santa Clara, CA, USA), which measured the enrichment of ^13^C (δ^13^C; parts per thousand difference from the standard) and the CO_2_ concentration produced by the bird's respiration. After the injection, a peak in the isotopic concentration occurred and a stable depletion followed (Fig. S1). After 5–10 min of stable isotopic washout, the bird was extracted from the chamber and was allowed to fly in the wind tunnel at a pre-set speed. After a flight of around 2 min (mean 2.1 min, range 1.8–2.4), the bird was captured and transferred back to the respirometry chamber, where isotope levels and CO_2_ production were again measured. Time stamps of each step were taken. All measurements were kept under 30 min to follow the assumption of single pool kinetics in small animals (Hambly and Voigt, 2011).

The pre- and post-flight δ^13^C data were natural log transformed and fitted with a linear regression. The regression slopes represent the log converted isotope elimination rate (*k*_c_; δ^13^C min^−1^) in the bird. We used the R-package *LolinR* (Olito et al., 2017) to select the best linear fit regression and avoid biases due to arbitrary data selection in the δ^13^C values. After fitting the linear regressions, pre- and post-flight *k_c_* were extrapolated until the time of flight start and flight termination, respectively, so a value of δ^13^C was inferred for the flight start and flight stop timestamps. The isotopic elimination rate during flight (flight *k*_c_) was calculated as the slope between the calculated pre- and post-flight δ^13^C values at the flight start and flight stop time stamps. As the isotope elimination rate is proportional to the amount of CO_2_ produced (Hambly and Voigt, 2011), flight CO_2_ was calculated by multiplying the flight isotopic elimination rate (flight *k*_c_) by the ratio between the post-flight CO_2_ production and the post-flight isotope elimination rate. This estimated flight CO_2_ production was then converted to *P*_met_ (W) following the protocol described in Macías-Torres et al. (2025).

All preliminary NaBi experiments (*n*=61) were visually inspected and any data showing unexpected patterns in the pre- and post-flight enrichment curves (i.e. a secondary enrichment in the isotope curve leading to a double peak), as well as irregularities in CO_2_ measurements were excluded from further analyses. In total, six measurements were discarded by this filtering. Furthermore, we assumed that a bird in flight consumes energy at a higher rate than in pre- and post-flight, during which birds remained mostly motionless inside the respirometry chamber. We therefore applied an additional criterion: estimated CO_2_ production during the flight had to be higher than the values directly measured before and after flight. Based on this criterion, nine samples were discarded. This resulted in a dataset of 46 NaBi experiments used to analyse horizontal flight *P*_met_. A second dataset consisted of nine NaBi samples collected from birds flying at a positive incline of 4 deg, which were used to calculate partial efficiency (see below).

### Outlier filtering

We screened for extreme values in flight *P*_met_ using the modified *Z*-score of Iglewicz and Hoaglin (1993), applied for binned airspeed centred at half-integers (*U*_0.5_). For each binned airspeed, we computed the bin median and the unscaled median absolute deviation (MAD):

(1) $$\mathrm{MAD}=\mathrm{media}n_{i}(|x_{i}-\tilde{x}|).$$

For each observation we then calculated the modified *Z*-score:

(2) $$M_{i}=(0.6745\times (x_{i}-\tilde{x}))/\mathrm{MAD}$$

and flagged observations as outliers when *M_i_*>3. Flagged observations were excluded from subsequent analyses. This screening was applied once and resulted in the removal of one outlier (2.92 W at 9 m s^−1^; *M_i_*=3.4); no further observations exceeded the threshold thereafter.

### Modelling power

After outlier removal (final *P*_met_ *n*=45 from 6 birds; *P*_mech_ *n*=27 from 6 birds), we fitted a model for flight *P*_met_ and *P*_mech_ as well as for the weight-specific flight power as a function of airspeed (*U*). We fitted flight *P*_met_ and *P*_mech_ and weight/lift-specific metabolic/mechanical power (*P*_met_^*^ and *P*_mech_^*^) with a simple linear and a quadratic polynomial in *U*. Models were fitted both with and without random intercepts for individual birds to control for the effect of between-bird variation. For *P*_mech_ and *P*_mech_^*^ models, the number of wingbeats was included as weighted in the model contributing to that estimate, assuming measurement precision increases with sample size. Model decisions were guided by fixed-effect significance, explained variance and the likelihood ratio test (see Supplementary Materials and Methods for details).

### Whole-animal energy conversion efficiency

Whole-animal energy conversion efficiency was calculated by dividing *P*_mech_ estimates by *P*_met_ estimates from the best fitted model at a given airspeed (*U*):

(3) $$\eta (U)=\frac{P_{\mathrm{mech}}(U)}{P_{\mathrm{met}}(U)}.$$

We then determined the maximum η(*U*) over the overlapping observed speed range (*U*) of the two datasets, η_max_, and its corresponding speed *U*_η*,*max_.

### Partial efficiency

We calculated the partial energy conversion efficiency (*η*_p_) during flight following the approach of Tucker (1972) and Bernstein et al. (1973). *η*_p_ was defined as the ratio between the mechanical power change (Δ*P*_mech_) attributable to a tunnel inclination (4 deg) and the corresponding change in metabolic power (Δ*P*_met_):

(4) $$\eta_{p}=\frac{\Delta P_{\mathrm{mech}}}{\Delta P_{\mathrm{met}}}\;,$$

where the mechanical power change was calculated as:

(5) $$\Delta P_{\mathrm{mech}}=W_{b}\;V=W_{b}\;\sin(\theta )\;U,$$

where *W*_b_*=m****g*** is the body weight, *m* is the mean body mass of all birds measured in kg (mean 0.0179 kg), ***g*** is the gravitational acceleration (9.81 m s^−2^), θ is the tunnel tilt angle (4 deg) measured with a clinometer, and *U* is the airspeed (11.4 m s^−1^).

We measured *P*_met_ during horizontal flight and climbing flight using the NaBi method (see above). Because the dataset consisted of limited samples per bird and some birds showed small or negative within-individual differences in *P*_met_ between horizontal and inclined flight, partial efficiency (η_p_) was estimated at the population level using a mixed-effects model. We fitted a linear mixed-effects model with flight power as the response variable, inclination as a fixed effect and a random intercept for bird individuals. The change in flight metabolic power (ΔP_met_) was estimated as the difference between the estimated marginal means in *P*_met_ flying at 0 deg and 4 deg (climbing flight) from the linear mixed model:

(6) $$\Delta P_{\mathrm{met}}={\bar{P}}_{\mathrm{met}\;\theta =4}-\;{\bar{P}}_{\mathrm{met}\;\theta =0}.$$

Uncertainty for η_p_, as the standard error (s.e.), was obtained using the delta method: s.e.(η_p_)=η_p_·[s.e.(Δ*P*_met_)/Δ*P*_met_]. The 95% CI was calculated as η_p_±1.96s.e.(η_p_).

### Muscle efficiency

We estimated flight muscle conversion efficiency as described in Morris et al. (2010). Flight muscle conversion efficiency was the ratio between *P*_mech_ output to *P*_met_ input, minus the basal metabolic rate (BMR). To account for overhead respiratory and circulatory costs, a factor of 1.1 was applied (Tucker, 1973). Hence, muscle efficiency (η_fm_) was calculated as:

(7) $$\eta_{\mathrm{fm}\;}(U)=\frac{1.1}{\left(P_{\mathrm{met}}(U)-\mathrm{BMR}\right)}\;P_{\mathrm{mech}}(U),\;$$

where *P*_met_ and *P*_mech_ are the metabolic and mechanical power estimates from the best-fitting models at a given airspeed in watts. BMR (W) represents the mean basal metabolic rate across individuals, predicted from body mass (*m*, in grams), using the allometric equation of McKechnie and Wolf (2004):

(8) log(BMR)=−1.461+0.669log(m).

In addition to the allometrically predicted BMR, we calculated flight muscle conversion efficiency by subtracting the mean post-flight recovery *P*_met_ (*P*_rec_, see below) from the flight *P*_met_:

(9) $$\eta_{\mathrm{fm},\mathrm{recovery}\;}(U)=\frac{P_{\mathrm{mech}}(U)}{\left(P_{\mathrm{met}}(U)-P_{\mathrm{rec}}\right)},$$

where *P*_rec_ is the mean post-flight recovery metabolic power across individuals (see below). Here, no overhead respiratory and circulatory factor was used as *P*_rec_ was measured while birds were performing these vital functions.

### *P*_met_ while not flying

In order to compare flight *P*_met_ with non-flight *P*_met_, we estimated the *P*_met_ of barn swallows while not in flight, during the recovery period right after a flight experiment. We refer to this measurement as post-flight recovery metabolic power (*P*_rec_). Following each trial, the bird was placed in a darkened respirometry chamber, and its metabolic rate was quantified from direct CO_2_ measurements using the isotope analyser system. The mean value of CO_2_ production rate was estimated from the best-fitting linear model using the R-package *LolinR* (Olito et al., 2017) and converted to *P*_met_ using the same calculation as applied to the flight power measurements.

We refer to this measurement as *P*_rec_, rather than resting metabolic rate (RMR), because the birds were not measured under standard resting conditions (Ellis and Gabrielsen, 2019). Specifically, the birds had been flying just a few seconds beforehand and had also been handled before being placed in the chamber. Thus, this value probably includes additional costs associated with recovery and handling stress.

We used the post-flight recovery phase rather than the pre-flight phase because pre-flight metabolic rates were consistently higher (as measured by CO_2_ production; Fig. S1), probably due to a higher stress level caused by longer handling, injection and the preparation period immediately before a flight experiment. In contrast, just before the post-flight phase, the birds were captured while flying and rapidly transferred to the respirometry chamber, which resulted in a lower metabolic rate during the post-flight than during the pre-flight phase. This is also the reason why the post-flight, rather than the pre-flight phase is used when estimating flight *P*_met_ (Elowe et al., 2023; Friman et al., 2024; Macías-Torres et al., 2025). Therefore, although post-flight *P*_rec_ should not be interpreted as true RMR because there may be some intrinsic stress involved, it provides the most conservative available individual estimate of *P*_met_ while not flying.

### Flight energetics in barn swallow versus thrush nightingale

We compared *P*_met_, *P*_mech_, *P*_met_^*^, *P*_mech_^*^ and energy conversion efficiency (η) between two species for which flight power was measured using the same methods in the same wind tunnel: barn swallows (this study) and thrush nightingales (Macías-Torres et al., 2025). We also compared the post-flight *P*_rec_ between the two species to evaluate differences in flight versus non-flight metabolism under the same circumstances in both species.

Additionally, cost of transport (COT) was calculated for each species considering both *P*_met_ and *P*_mech_. COT is the energy expenditure per unit weight over unit distance. It was calculated as:

(10) $$\mathrm{COT}=\frac{\mathrm{Power}}{W\times U},$$

where Power is *P*_met_ or *P*_mech_ in watts (J s^−1^), *W* is the weight in newtons and *U* is the flight speed in m s^−1^. Thus, COT is a dimensionless number.

### Statistical analysis

Uncertainty in η_max_, *U*_η*,*max_ and η_fm​_ was quantified using a non-parametric case bootstrap (bootstrap iterations=50,000). For each replicate, we resampled rows independently within the mechanical and metabolic datasets (sampling with replacement), refitted both quadratic models and recomputed the efficiency calculation. We report bootstrap means and 95th percentile confidence intervals (CI).

Additionally, uncertainty in η_p_ was obtained via a stratified non-parametric bootstrap: we resampled metabolic flight power within each inclination separately with replacement preserving the original sample sizes (bootstrap iterations=50,000). This resampling draws samples from the empirical distribution of the observed data, so that an observation can appear multiple times. For each replicate, we computed the bootstrapped mean difference Δ*P*_met_^(*b*)^ and then calculated η_p_^(*b*)^=Δ*P*_mech_/Δ*P*_met_^(*b*)^ while keeping Δ*P*_mech_ fixed from the previously calculated value. We report the 95% CI from the bootstrap analysis.

Uncertainty in minimum power speed (*U*_mp_) and its corresponding flight power (*P*_mp_) was quantified via a non-parametric case bootstrap. We drew *B*=5000 bootstrap samples by resampling rows of the *P*_met_ and *P*_mech_ dataset with replacement (preserving sample size), refitted the same quadratic model to each resample, and recomputed *U*_mp_ and *P*_mp_. Because a meaningful minimum requires a convex parabola, bootstrap fits with *b*^2^≤0 were discarded and excluded from summaries; we report the proportion of such replicates. For valid replicates, we summarised uncertainty using the bootstrap 95% CI for both *U*_mp_ and *P*_mp_.

Analyses were performed in R studio (v4.5.0). Mixed-effects models were fitted with lme4 (*lmer*) and *P*-values/d.f. obtained via *lmerTest* (Bates et al., 2015). Estimated marginal means and contrasts (Δ*P*_met_) were computed with *emmeans* (https://cran.r-project.org/package=emmeans). From all the fitted models, the best-performing one was a simple polynomial model without random effects, as including individual as a random intercept resulted in model singularity (Supplementary Materials and Methods).

## RESULTS

### *P*_met_

**Fig. 1.**
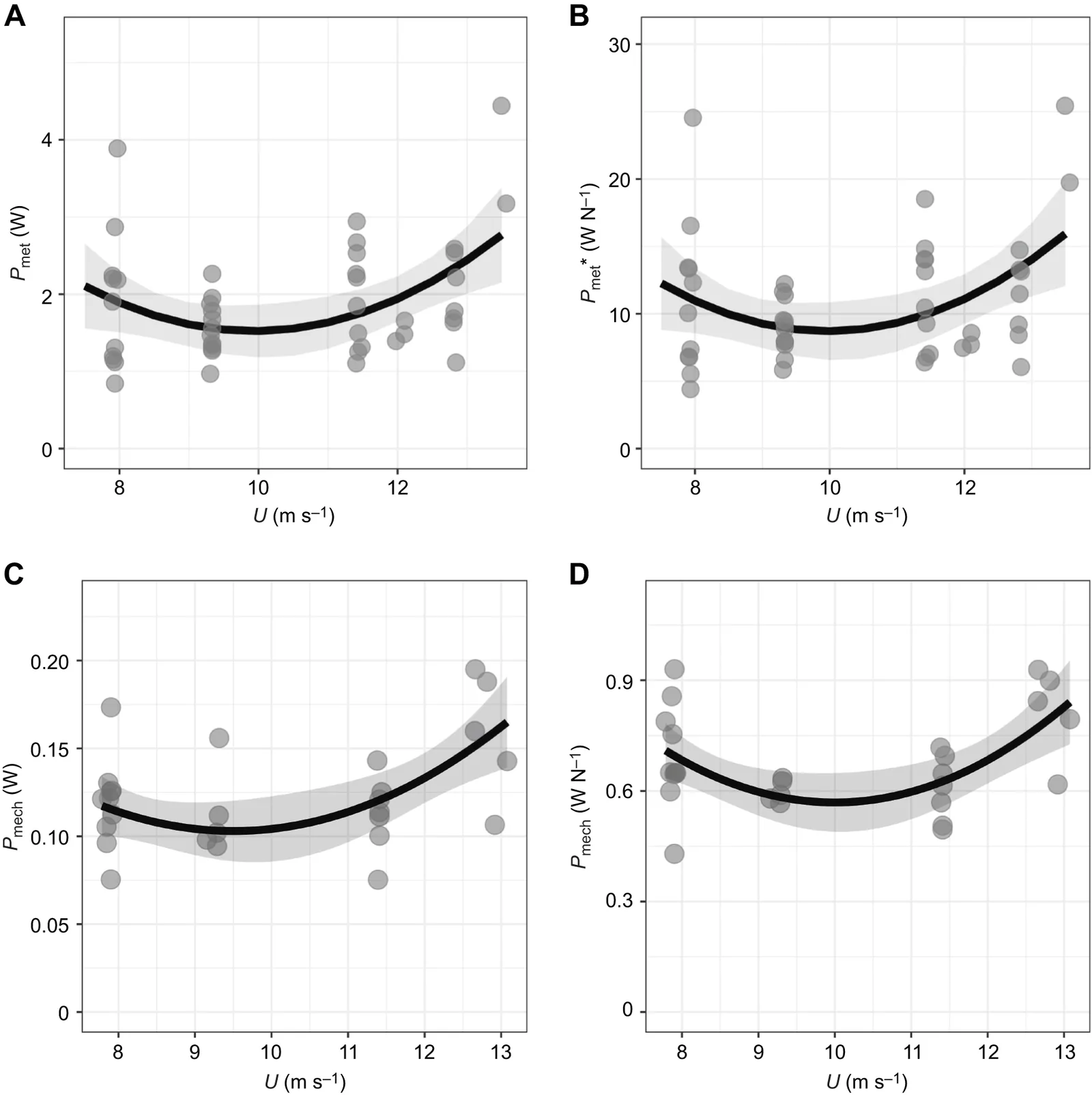
**Power versus air speed in barn swallows (*Hirundo rustica*) flying in a wind tunnel.** (A) Metabolic power *P*_met_=11.21–1.95*U*+0.098*U*^2^ and (B) weight-specific metabolic power *P*_met_^*^=66.44–11.58*U*+0.58*U*^2^ (where *U* is airspeed). (C) Mechanical power *P*_mech_=0.54–0.092*U*+0.0048*U*^2^ and (D) lift-specific mechanical power *P*_mech_^*^=3.35–0.566*U*+0.0287*U*^2^. Grey circles show individual measurements, while black lines indicate quadratic polynomial fits with 95% confidence intervals (CI; shaded areas).

*P*_met_ was 1.52 W (bootstrap mean: 1.49 W; 95% CI: 1.23–1.77 W) at *U*_mp_=9.9 m s^−1^ (bootstrap mean: 9.6 m s^−1^; 95% CI: 7.4–10.7 m s^−1^), while *P*_met_^*^ was 8.72 W N^−1^ (bootstrap mean: 8.49 W N^−1^; 95% CI: 6.83–10.19 W N^−1^) at *U*_mp_=9.97 m s^−1^ (bootstrap mean: 9.63 m s^−1^; 95% CI: 7.5–10.69 m s^−1^) (Fig. 1).

### *P*_mech_

*P*_mech_ was 0.10 W (bootstrap mean: 0.10 W; 95% CI: 0.08–0.12 W) at *U*_mp_=9.5 m s^−1^ (bootstrap mean: 9.4 m s^−1^; 95% CI: 7.5–10.3 m s^−1^), while *P*_mech_^*^ was 0.57 W N^−1^ (bootstrap mean: 0.57 W N^−1^; 95% CI: 0.51–0.61 W) at *U*_mp_=10 m s^−1^ (bootstrap mean: 10 m s^−1^; 95% CI: 9.4–10.5 m s^−1^) (Fig. 1).

### Whole-animal energy conversion efficiency (η)

**Fig. 2.**
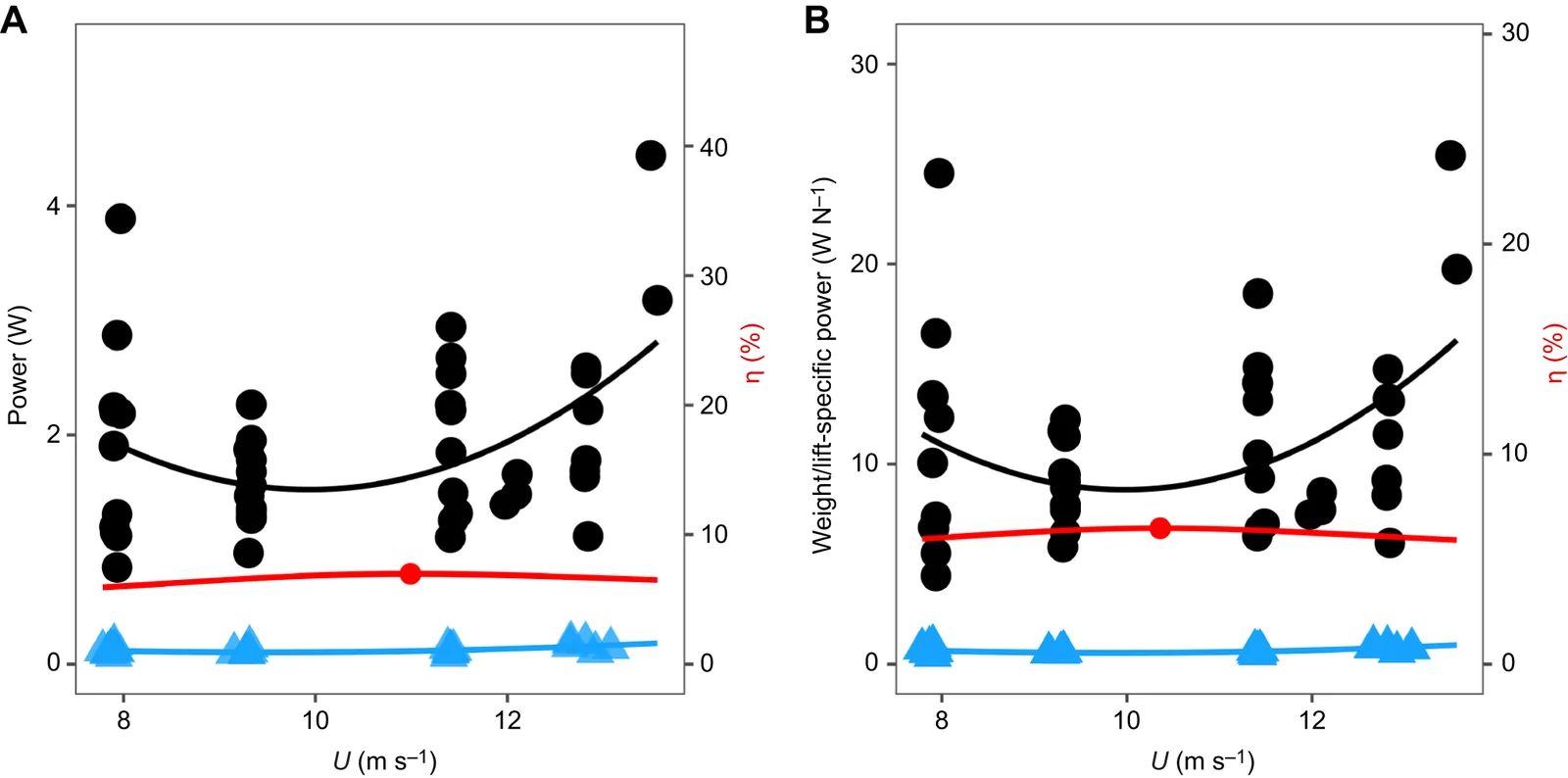
**Flight power and whole-animal conversion efficiency versus airspeed in barn swallows (*H. rustica*) flying in a wind tunnel.** (A) *P*_met_ and *P*_mech_, and whole-animal energy conversion efficiency (η). (B) *P*_met_^*^ and *P*_mech_^*^, and η. Black circles represent metabolic power; blue triangles represent mechanical power; red lines represent the whole-animal conversion efficiency calculated by dividing the lines fitted to the mechanical and metabolic power in each panel. The red circle denotes the peak efficiency and the speed it occurs at.

Whole-animal energy conversion efficiency η for raw values of *P*_met_ and *P*_mech_ resulted in a peak of 7% (bootstrap mean: 7.7%; 95% CI: 6.4–9.6%) at 11 m s^−1^ (bootstrap mean: 10.8 m s^−1^; 95% CI: 7.9–13.1 m s^−1^). Whole-animal energy conversion efficiency η based on *P*_met_^*^ and *P*_mech_^*^ showed a peak of 6.5% (bootstrap mean: 7.3%; 95% CI: 6–9.6%) at 10.4 m s^−1^ (bootstrap mean: 10.4 m s^−1^; 95% CI: 7.9–13.6 m s^−1^) (Fig. 2, Table 1).

**Table 1.** Energy conversion efficiency estimates calculated in this study

	Maximum value (95% CI)	Airspeed (*U*) at maximum value, η_max_ (95% CI)
Whole-animal energy conversion efficiency (η)	7% (6–9.6)	11 m s^−1^ (7.9–13.1)
Muscle conversion efficiency (η_fm_)	9% (7.1–11.6)^*^	10 m s^−1^
	12.7% (9.1–19.5)^‡^	10 m s^−1^
Partial conversion efficiency^§^ (η_p_)	27% (delta method: −2–56.1; bootstrapping: 12–153.8)	11.4 m s^−1^ (only one airspeed measured)

Whole-body energy conversion efficiency is derived from the best-fitting models for mechanical and metabolic power. ^*^Muscle conversion efficiency was calculated based on Morris and Askew (2010). ^‡^Muscle conversion efficiency was calculated relative to the metabolic rate when the barn swallows were not flying (post-flight recovery metabolic power, *P*_rec_; see Materials and Methods). ^§^Partial conversion efficiency was estimated based on the tilted wind tunnel method (Bernstein et al., 1973; Tucker, 1972). CI, confidence interval.

### Partial efficiency (η_p_)

Flight *P*_met_ was measured during horizontal flight (0 deg) and a positive climb angle (4 deg) at 11.4 m s^−1^. We analysed 20 flights from 6 birds, 11 horizontal and 9 climbing flights (Table S1). The mixed model indicated a positive, marginal effect of inclination on flight *P*_met_: estimate=0.13 W, s.e.=0.07 W (*t*_16.49_=1.95*t*, *P*=0.068). Expressed as 4 deg−0 deg values, the estimated marginal mean difference in metabolic power (Δ*P*_met_) was 0.52 W (s.e.=0.28 W; Fig. S2). The calculated change in *P*_mech_ due to a climbing flight of 4 deg (Δ*P*_mech_) was 0.14 W. This resulted in a η_p_ of 27% (delta-method 95% CI: −2–56%). The non-parametric bootstrapping yielded a median of Δ*P*_met_^(*b*)^=0.53 W with a 95% CI: 0.02–1 W; bootstrap median η_p_=26% and 95% CI: 12–154%.

### Flight muscle efficiency (η_fm_)

**Fig. 3.**
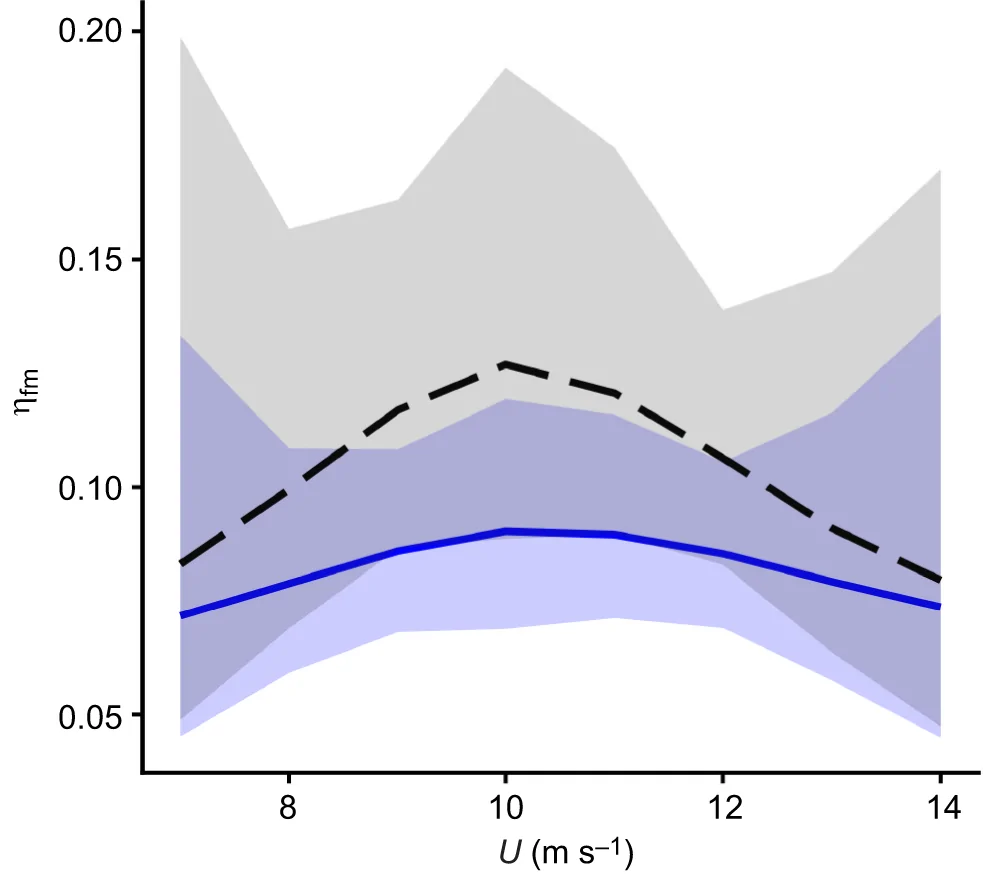
**Flight muscle efficiency estimates in barn swallows (*H. rustica*).** Flight muscle conversion efficiency η_fm_=1.1×*P*_mech_/(*P*_met_−BMR) (following Morris et al., 2010) (blue); and recovery η_fm,recovery_=*P*_mech_/(*P*_met_−*P*_rec_) (black dashed), across flight speeds from 7 to 14 m s^−1^, where *P*_rec_ is the metabolic power after a flight experiment, during recovery (see Materials and Methods), and BMR is basal metabolic rate. The lines represent the estimated mean values from bootstrapping methods; shaded areas show bootstrapping 95% CI for each corresponding approach.

Muscle efficiency calculations resulted in a maximum of 9% (bootstrapping 95% CI: 7.1–11.6%) at 11 m s^−1^, showing a shallow concave function in the mean values (Fig. 3, Table 1).

### Flight versus non-flight *P*_met_

Mean *P*_rec_ was on average 0.7 W (s.e.=0.01 W) for barn swallows. Metabolic flight cost at *U*_mp_ was 2.19 times more costly than *P*_rec_. Estimated BMR from allometric equations was 0.24 W (s.e.=0.002 W). Thus, metabolic flight cost at *U*_mp_ was 6.4 times more costly than estimated BMR.

### Barn swallow versus thrush nightingale

**Fig. 4.**
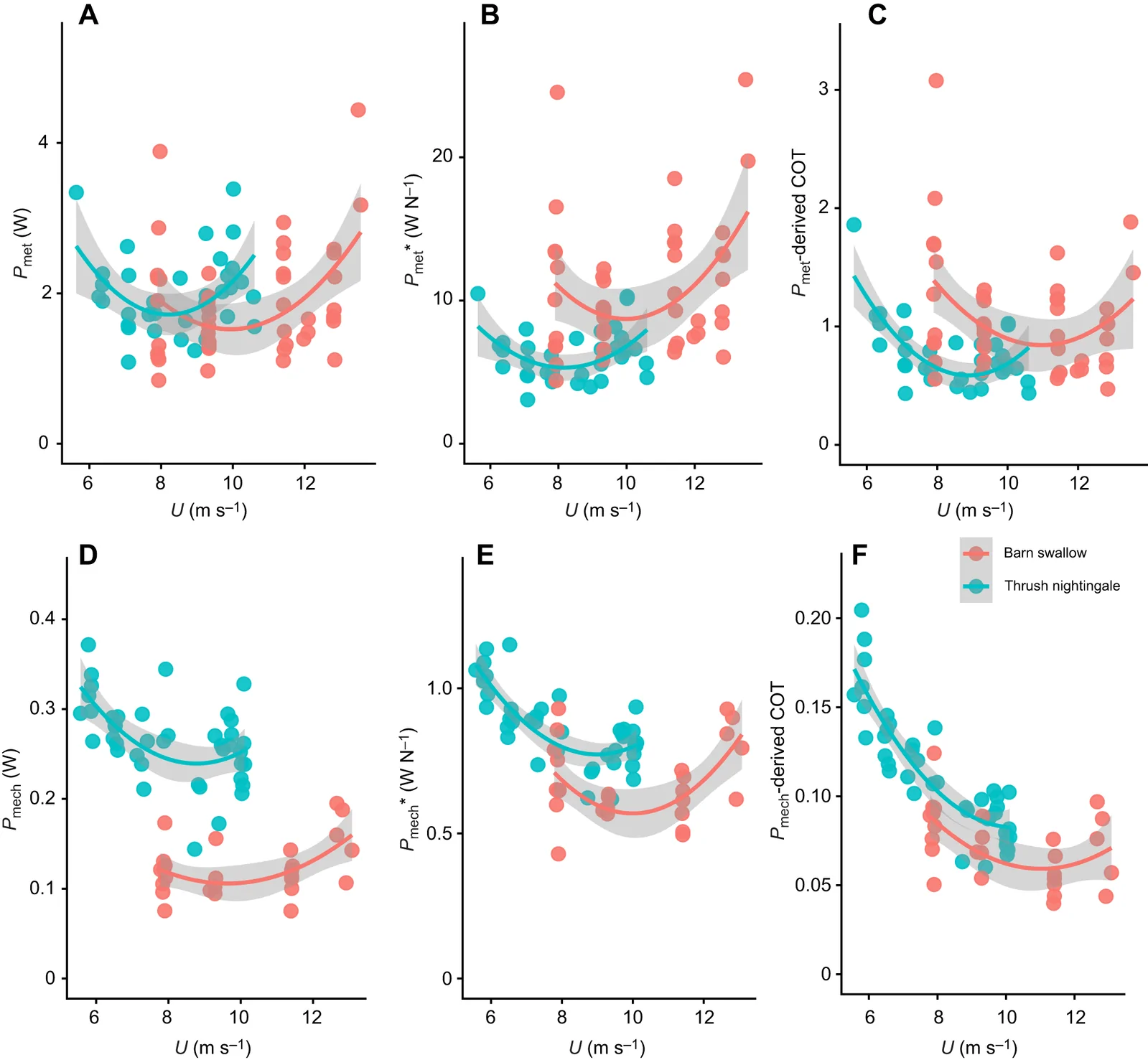
**Comparison of flight metabolic and mechanical power between barn swallows and thrush nightingales across airspeeds.** (A) *P*_met_, (B) *P*_met_^*^, (C) cost of transport (COT) based on *P*_met_, (D) *P*_mech_, (E) *P*_mech_^*^ and (F) COT based on *P*_mech_ plotted against airspeed (*U*). COT (dimensionless) is the ratio between the flight power (metabolic or mechanical) and the bird's weight (N) multiplied by the flight speed. Coloured lines are the best-fitting polynomial models, and the grey shading is the standard error of the model.

Mean *P*_met_ did not differ significantly between barn swallows (mean 1.89 W) and thrush nightingales (mean 2.01 W; Welch two-sample *t*-test: *t*=−0.82, d.f.=76.7, *P*=0.41; 95% CI: −0.41 to 0.17; Fig. 4A). In contrast, mean *P*_met_^*^ was significantly higher in barn swallows (mean 10.9 W N^−1^) than in thrush nightingales (mean 6.3 W N^−1^; Welch two-sample *t*-test: *t*=6.04, d.f.=59.5, *P*<0.0001; 95% CI: 3.07 to 6.12; Fig. 4B).

*P*_mech_ differed significantly between the two species. Barn swallows showed lower mean *P*_mech_ (0.12 W) compared with thrush nightingales (0.27 W; Welch two-sample *t*-test: *t*=−15.53, d.f.=65.9, *P*<0.0001; 95% CI: −0.16 to −0.12; Fig. 4D). Additionally, *P*_mech_^*^ was significantly lower in barn swallows (mean 0.68 W N^−1^) than in thrush nightingales (mean 0.86 W N^−1^; Welch two-sample *t*-test: *t*=−5.82, d.f.=53.8, *P*<0.0001; 95% CI: −0.25 to −0.12; Fig. 4E).

Average *P*_met_-derived COT across all flight speeds was higher in barn swallows than in thrush nightingales, with a mean *P*_met_-derived COT of 1.1 (interquartile range IQR: 0.71–1.25) compared with a *P*_met_-derived COT of 0.78 (IQR: 0.61–0.86) in thrush nightingales (Fig. 4C). The opposite pattern was observed for the *P*_mech_-derived COT across all flight speeds, where thrush nightingales had a mean *P*_mech_-derived COT of 0.11 (IQR: 0.09–0.13); barn swallows mean *P*_mech_-derived COT was 0.07 (IQR: 0.06–0.9; Fig. 4F).

Post-flight recovery metabolic power (*P*_rec_; calculated from the measured amount of CO_2_ produced after performing an experimental flight) was significantly lower in barn swallows (mean 0.7 W) than in thrush nightingales (mean 0.82 W; Welch two-sample *t*-test: *t*=−8.05, d.f.=77.0, *P*<0.0001; 95% CI: −0.15 to −0.09). However, when accounting for body mass, weight-specific *P*_rec_ was significantly higher in barn swallows (mean 4 W N^−1^) compared with thrush nightingales (mean 2.5 W N^−1^; Welch two-sample *t*-test: *t*=16.61, d.f.=61.6, *P*<0.0001; 95% CI: 1.3 to 1.6; Fig. S3).

## DISCUSSION

Our wind-tunnel measurements provide direct estimates of *P*_met_ and *P*_mech_ in un-instrumented flying barn swallows. As expected, both *P*_met_ and *P*_mech_ followed classic U-shaped speed–power curves (Fig. 1). We observed that whole-animal conversion efficiency η had a shallow peak at ∼7% at a speed of 11 m s^−1^, which is lower than direct estimates in other passerines species (Hedh et al., 2020; Macías-Torres et al., 2025), while consistent with the predicted body-mass scaling of conversion efficiency (Currie et al., 2023; Groom et al., 2018). Our results indicate that barn swallows, although less efficient fliers than hypothesised, have a wider range of speeds at which they can fly while performing relatively well. This pattern suggests that flight energy conversion efficiency is both species and speed dependent, and it is probably tuned to match the range of ecologically relevant speeds used by each species.

### Power measurement validation

Barn swallow *P*_met_ has been estimated previously using different methods, resulting in a range of values: 1.34 W (mass loss; Lyuleeva, 1970), 1.3 W (doubly labelled water; Hails, 1979) and 1.62 W (doubly labelled water; Turner, 1982). These measurements were performed on freely flying birds in the wild, and thus the flight speed is unknown, but it is expected that the birds flew at speeds between minimum power and maximum range speeds (Hedenström and Alerstam, 1995). Thus, the measurements of free flying barn swallows agree well with the measured values in the wind tunnel in this study (*P*_met_ at *U*_mp_ 1.52 W). Another wind tunnel study, using doubly labelled water to measure *P*_met_ in barn swallows, yielded a value of 2.13 W (range 1.74–2.55 W, s.d. 0.21 W at 10.3 m s^−1^; Schmidt-Wellenburg et al., 2007). Additionally, *P*_met_ can be estimated from allometric equations, such as in Bishop and Butler (2015): *P*_met_=52.6*M*^0.74^, where *M* is mass (kg). This model predicts a *P*_met_ of 2.69 W for a bird of mass 18 g. Our measured *P*_met_ at *U*_mp_ (1.52 W) lies within the range of previous measurements and estimates, suggesting that our measurements are realistic. Additionally, it is worth noting that our study birds were all juveniles, and we cannot exclude that flight metabolic costs may be slightly higher than would be expected in fully mature adults. This is because juvenile birds may still be refining flight behaviour, neuromuscular control and flight-related morphology (see Ruaux et al., 2023).

*P*_mech_ measurements were estimated in barn swallows in a previous study performed in the same wind tunnel with values ranging from 0.17 to 0.27 W across a range of speeds between 6 and 11 m s^−1^ for a 19 g individual (Pennycuick et al., 2000). *P*_mech_ values previously reported were obtained using a different method from that in this study: kinematics of the body and humerus were recorded with high-speed video, and these data were then combined with aerodynamic and inertial principles to calculate the forces, moments and angular displacements, from which *P*_mech_ output was derived (Pennycuick et al., 2000). They report that their *P*_mech_ measurements ‘turned out to be more than that predicted on the basis of current estimates of body drag coefficient and profile power ratio, possibly because the bird was not flying steadily in a minimum-drag configuration’. Thus, our *P*_mech_ measurements (0.07–0.19 W), although lower than previously measured, seem to be realistic. We acknowledge that wake dissipation may have reduced the measured wake energy relative to the energy present immediately behind the wings. However, because measurements were made relatively close to the birds, this effect is likely to be small. Moreover, any dissipation-related underestimation should be broadly consistent across speeds and comparable to that in previous *P*_mech_ values reported under the same experimental approach (Currie et al., 2023; Hedh et al., 2020; Johansson et al., 2018; Macías-Torres et al., 2025).

In this study, *P*_mech_ was calculated as the wake energy per wingbeat multiplied by the wingbeat frequency of the analysed sequence, i.e. the rate at which energy is added to the wake during the analysed sequence. Because our *P*_mech_ measurements were based on active flapping wingbeats only, they may overestimate the real wingbeat frequency of the species. To compare our values with realistic wingbeat frequencies (effective wingbeat frequencies that include intermittent pauses between wingbeats, *F*_eff_), we compared our values with *F*_eff_ from Bruderer et al. (2001) for barn swallows in horizontal flight. Across the same airspeeds as our PIV measurements, the estimated *F*_eff_ from Bruderer et al. (2001) averaged 6.76±0.30 Hz, whereas wingbeat frequency measured during our PIV sequences averaged 8.86±1.09 Hz (Fig. S4). Thus, observed wingbeat frequencies during active sequences were on average 2.10 Hz higher, corresponding to approximately 31% greater values than the effective wingbeat frequencies estimated from Bruderer et al. (2001).

Consequently, our *P*_mech_ estimates represent active-flapping *P*_mech_ and may overestimate the time-averaged *P*_mech_ over complete flight cycles that include pauses. Additionally, wingbeat frequency and wake energy are not independent, especially if the active wingbeats compensate for the mid-upstroke pauses observed in this species. If that is the case, the energy added to the wake per active wingbeat may be higher than it would be during continuous flapping without pauses. So, if active wingbeats have both higher frequency and higher energy per beat, estimated *P*_mech_ should be potentially higher than for continuous flapping flight. Furthermore, simply replacing the measured wingbeat frequency with *F*_eff_, while keeping the measured wake energy per wingbeat unchanged, would not provide a realistic estimate of time-averaged *P*_mech_. Instead, it would assume that each active wingbeat generates the same wake energy regardless of whether pauses are included, which is unlikely, in addition to the unknown power generated during the pauses only. Consequently, our *P*_mech_ measurements are probably an overestimation of true flight *P*_mech_ of the flap-gliding flight style of barn swallows flying in a wind tunnel.

### Whole-animal energy conversion efficiency

Barn swallows showed a shallow peak in conversion efficiency at intermediate speeds, consistent with observations from another species (thrush nightingales) studied using the same methodology, although this peak was not sharply defined (cf. Macías-Torres et al., 2025). Instead, the bootstrapping method indicates that barn swallows have a broad range of speeds at which they can fly while performing close to their peak efficiency, spanning much of the tested speed range. Being similarly efficient at a wide range of flight speeds probably benefits aerial insectivores such as barn swallows, which exploit a wide speed range during prey capture (Turner, 1982).

The flight conversion efficiency value in barn swallows in this study was roughly half of previous estimates for thrush nightingales (15%; average mass 33 g; Macías-Torres et al., 2025) and blackcaps (*Sylvia atricapilla*; 14–22%; average mass 19 g; Hedh et al., 2020), suggesting that barn swallows are less efficient overall. This pattern is consistent with the body-mass scaling of conversion efficiency, which predicts that smaller birds are less efficient than larger ones (Bishop, 2005; Currie et al., 2023; Groom et al., 2018). Applying the mass scaling equation by Currie et al. (2023) to barn swallows (body mass 17.9 g) yields a predicted whole-animal conversion efficiency of 6.2% (s.d. 0.1), which aligns well with our measurements, providing support for our findings. By contrast, using the same mass scaling equation for thrush nightingales predicts an efficiency of ∼7.2%, well below the 15% in Macías-Torres et al. (2025). This discrepancy between mass-scaling predictions and measured conversion efficiency emphasises the need for additional cross-species measurements of energy conversion efficiency. Additionally, our support for a body-mass scaling of efficiency (Currie et al., 2023) offers a mechanistic explanation for why intermediate size birds, and not the smallest birds, perform the longest non-stop migrations, as seen in several wader species (Gill et al., 2009; Hedenström, 2010; Klaassen et al., 2011).

Barn swallows in this study performed intermittent flap-gliding flight, alternating active flapping phases with fixed-wing bounds in the middle of the upstroke (Bruderer et al., 2001). The gliding phase can provide support for up to 20% of the body weight as lift (Tobalske, 2010, 2022). These ‘fixed-wing’, partly outstretched wings, gliding phases were excluded from our *P*_mech_ calculations (consisting of just 2–6 wingbeats), but were included in the *P*_met_ estimates, as these lasted about 2 min. Consequently, our *P*_mech_ probably reflects an upper bound relative to the time-averaged costs of free flight. If our measurements overestimate the *P*_mech_ of barn swallows, then flight energy conversion efficiency would be even lower than the values calculated here. Similarly, an overestimation of *P*_met_ due to handling stress is also a possibility; however, published values for freely flying barn swallows (Hails, 1979; Lyuleeva, 1970; Turner, 1982) align well with our *P*_met_ measurements. Finally, it is possible that barn swallows incorporate some energy-saving flight behaviours during migration, such as using fixed-wing bounds (Tobalske, 2022) or by exploiting wind-speed gradients while gliding with fully extended wings (Warrick et al., 2016). Such behaviours could reduce the overall power required for sustained flight compared with continuous flapping.

Yet another possibility is that the natural flight style of barn swallows, with frequent and rapid manoeuvres, with pauses in the middle of the upstroke sequence (Bruderer et al., 2001), prevents them from using energy-saving adaptations suggested for other birds. For example, it has been shown that birds can store energy in the tendon of the supracoracoideus muscle when continuously flapping, allowing them to operate at higher efficiency (e.g. Deetjen et al., 2024). However, the flap-gliding flight seen in our swallows may require active breaking of the wingbeat sequence and overcoming higher inertial power than in other birds (or during steady migratory flight). Similarly, muscle incurs energetic costs not only when producing mechanical work but also during activation, force development and relaxation, with brief downstroke pauses and reactivation possibly increasing the relative cost of muscle contraction compared with the time available for positive mechanical work (Usherwood, 2016). Thus, a muscle activation cost from resuming multiple wingbeats after pauses in barn swallows may result in a relatively high *P*_met_, partly explaining the apparently low energy conversion efficiency. At the same time, this may not be reflected in the aerodynamic power to the same degree, as *P*_mech_ is largely determined by the aerodynamic properties of the wings and body.

Lastly, barn swallows often showed lateral movement in the wind tunnel, moving back and forth between the sides of the test section, ‘surfing’, rather than maintaining a completely steady position. Such behaviour may increase *P*_met_ because it involves repeated adjustment in body orientation, force production and flight control, all involving muscle activation. However, these costs may not be fully reflected in *P*_mech_, which was calculated from the wake during selected relatively stable flight sequences and therefore mainly represents the aerodynamic power of the analysed wingbeats. This mismatch between whole-animal metabolic costs and locally measured aerodynamic power could contribute to the relatively low apparent energy conversion efficiency. We stress, however, that these lateral movements were not equivalent to the rapid manoeuvres barn swallows perform in the field, but may represent an additional cost relative to the steadier flight of species previously measured under similar conditions.

### Partial and muscle efficiency

The first ever estimate of partial efficiency (η_p_) resulted in a mean value of 23% (Bernstein et al., 1973; Tucker, 1972) and has been used as the reference value in flight power models ever since (Pennycuick, 1975, 2008). η_p_ is the ratio of the change in power output to power input at different flight efforts: horizontal flight versus climbing flight. The partial efficiency is thus independent of whatever basal metabolism the bird has, assuming that the basal metabolism remains the same at the two climbing rates. Thus, it relates to the flight engine efficiency solely and should reflect similar values to those of flight muscle efficiency.

Mean η_p_ measured here (27%) resembles the values previously obtained, although the uncertainty in our estimate limits its reliability. Flight muscle efficiency estimated in this study only increased the efficiency by a small degree compared with whole-animal conversion efficiency, reaching a maximum of 12.7% at intermediate speeds, aligned with the maximum whole-animal efficiency speed. The discrepancy between partial and muscle/whole-animal efficiency should be interpreted with caution, as partial efficiency estimates were less reliable.

### Flying in a wind tunnel

In this study, barn swallows were restricted to fly in the confined space of a wind tunnel, which inevitably constrains their natural flight behaviour, as they cannot fully exploit the surrounding air to gain kinetic or potential energy as they would do under natural conditions (Masman and Klaassen, 1987). Barn swallows are capable of a diverse flight repertoire in the wild, including gliding, partial bounding, exploitation of near-ground wind gradients during manoeuvres, and reducing wingbeat frequency to capitalise on wind speed gradients close to the surface (Warrick et al., 2016). More broadly, many diurnal migrants shift between continuous flapping, intermittent flight and soaring to reduce energetic costs (Hedenström, 1993; Sapir et al., 2010; Shamoun-Baranes et al., 2016; Tobalske, 2010), behaviours that are inextricably linked to the limitations of flying in a wind tunnel. The range of airspeeds recorded for wild-flying barn swallows spans from 3.7 to 19.4 m s^−1^, with a mean of 9.5±2.7 m s^−1^ (Warrick et al., 2016), which overlaps with the flight speeds measured in this study. Notably, the mean wild flight speed aligns with the minimum power speed identified in this study, suggesting comparable baseline flight performance despite experimental constraints. Thus, we believe that flight power estimates from wind tunnel flights in this study provide valuable insights into the baseline of barn swallow flight energetics, although they may not fully capture the full range of flight flexibility that barn swallows make use of in the wild.

### Flight power in barn swallows versus thrush nightingales

Flight *P*_met_ and *P*_mech_ is hard to measure in free flying birds and usually comparisons are based on studies using various methodologies (Bishop and Guglielmo, 2022; Tobalske et al., 2003). Here, we discuss the flight power results obtained for two passerine species measured under the same circumstances and using the same methodology. We showed that the two species differed in the range of flight speeds that they could fly at, with species-specific U-shaped power curves (Fig. 4).

*P*_met_ differed between the two species, with weight-specific flight *P*_met_ being higher in barn swallows than in thrush nightingales (Fig. 4B), supporting the hypothesis that flight power scales with body mass with an exponent less than 1 (Bishop and Butler, 2015; Hedenström, 2010). Additionally, smaller birds have higher BMR in proportion to their mass (Lasiewski and Dawson, 1967), resulting in their cost of flight being a smaller multiple of their BMR than otherwise expected. This was not fully supported by our measurements as the flight *P*_met_ was a similar multiple of their estimated BMR from allometric equations (in barn swallows, flight *P*_met_ at *U*_mp_ was 6.4×BMR, while in thrush nightingales it was 6.1×BMR).

The post-flight recovery metabolic rate (inferred from measured CO_2_) is sampled a few minutes after the bird's flight experiment and probably reflects values related to the field metabolic rate, when not performing any flight. This study's post-flight recovery metabolic rates (mean±s.e.m. 4±0.08 W N^−1^) were 33% lower than field metabolic rates measured in the same species during an entire day using doubly labelled water (104 kJ day^−1^ for a 20.4 g bird, equivalent to 6 W N^−1^; Nagy, 1987). Absolute post-flight recovery metabolic rate was lower in barn swallows than in thrush nightingales, as expected for a bird with lower mass. However, weight-specific post-flight recovery metabolic rate was higher in barn swallows than in thrush nightingales, as expected in smaller birds showing higher relative metabolism (Lasiewski and Dawson, 1967). This resulted in barn swallows having almost the same flight power if compared with their post-flight metabolic rate as in thrush nightingales (in barn swallows flight *P*_met_ at *U*_mp_ is 2.19 times post-flight *P*_rec_, while in thrush nightingales it is 2.07 times post-flight *P*_rec_). All this indicates that barn swallows have a higher background metabolic rate while flying compared with thrush nightingales, which supports our finding of barn swallows showing a lower whole-animal energy conversion efficiency than thrush nightingales.

*P*_mech_^*^ illustrates that barn swallows fly at a lower cost per unit of lift than thrush nightingales, suggesting a higher aerodynamic efficiency in barn swallows compared with the thrush nightingales (Fig. 4D). One cause of this better aerodynamic performance could be a lower body drag, due to a more streamlined body shape, or a more efficient lift production due to the wings having a higher aspect ratio. This was noted in the first wind tunnel studies in this species (Pennycuick et al., 2000).

### Conclusion

Overall, our results emphasise the importance of accounting for scaling when considering conversion efficiency in flying animals. This becomes particularly important when using flight models, such as ‘afpt’ (Klein Heerenbrink et al., 2015; Tools for Modelling of Animal Flight Performance: https://cran.r-project.org/package=afpt), to make predictions about flight energetics. In addition, the difference we found between our barn swallows and previously studied species (i.e. thrush nightingale; Macías-Torres et al., 2025) points towards not only species-specific optimal (i.e. peak efficiency) flight speeds but also ecologically derived variation in the shape of the efficiency curve across flight speeds. While barn swallows may benefit from a wide range of flight speeds that they can efficiently fly at, thrush nightingales perform intensive continuous flights only during migration and may benefit from having a narrower range of speeds with higher efficiency. This introduces the idea that there might be a trade-off between specialisation and flexibility, with higher efficiency at a narrow speed range versus lower efficiency spread across a broader range. However, few species have been studied thus far, and more studies are needed to draw firm conclusions on how ecology, physiology or other factors (e.g. phylogeny) shape the conversion efficiency of a species.

## Supplementary Material

10.1242/jexbio.252390_sup1 Supplementary information
