# Supplementary material for: Flight energetics of barn swallows across speeds: high aerodynamic efficiency, but low energy conversion efficiency
Source: J Exp Biol. 2026 Sep 17;229(18):jeb252390. doi: 10.1242/jeb.252390 (PMC13615695; doi:10.1242/jeb.252390)
Supplement: Supplementary file 1 [file jexbio-229-252390-s1.pdf]

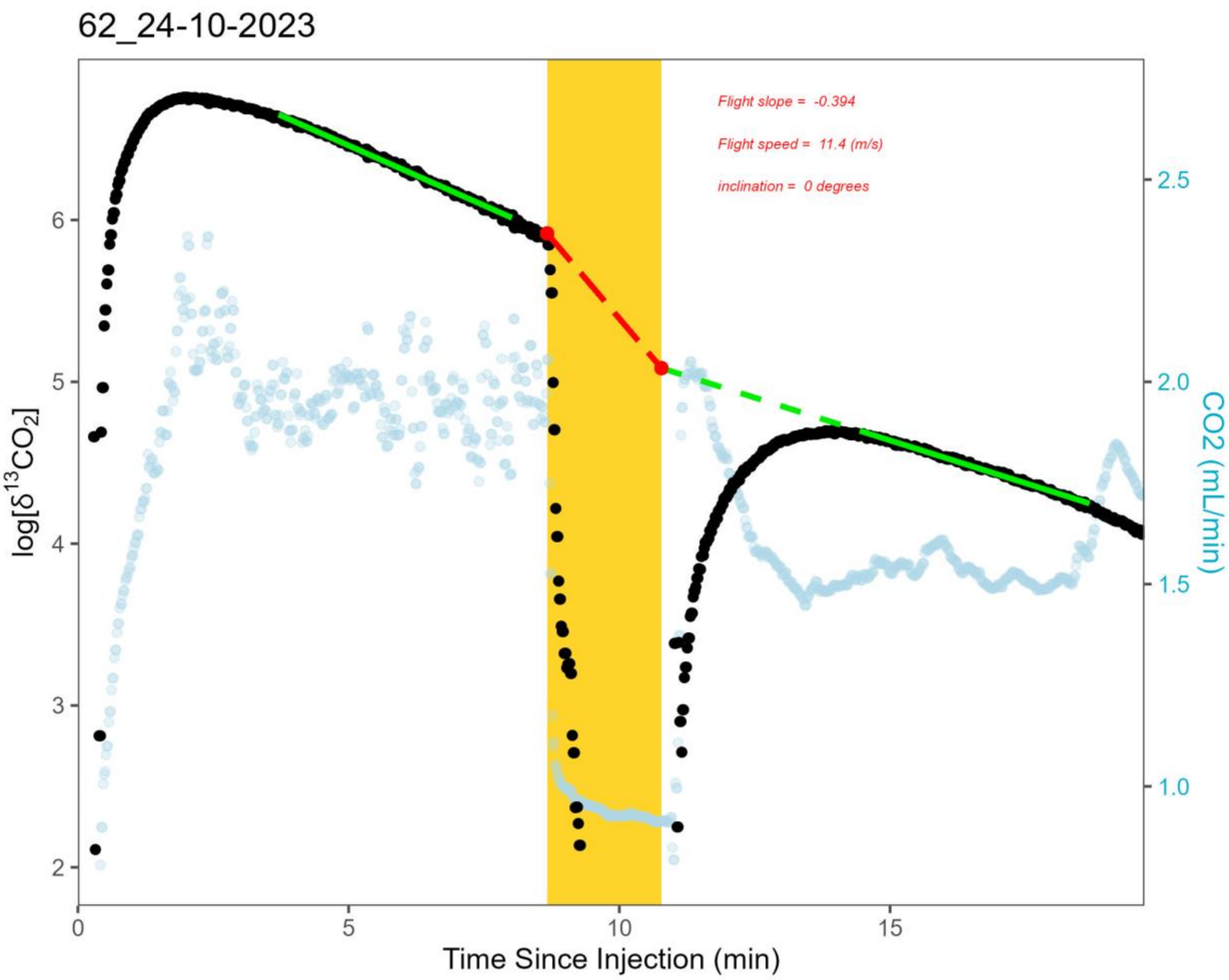

Fig. S1. Metabolic power measurement example using the ^{13}C NaBi technique in barn swallow (*H. rustica*). Figure represents the data sampled with the isotope analyser from the respirometry chamber airstream. Black dots are natural log-transformed measured values of $\delta^{13}C$ (left y axis). Light blue dots are measured values of CO_2 (right y axis). The yellow rectangle indicates the flight time of a barn swallow inside the wind tunnel. Green lines are the best-fitting linear regression for the pre- and post-flight periods (before and after the yellow rectangle, respectively). The red dashed line is the result extrapolating pre- and post-flight linear regressions until the beginning and the end of the flight (dashed green line). This red slope indicates the ^{13}C isotope elimination rate during the flight experiment. The red values indicate the resulting red slope during the flight period, the calculated VCO_2, and the wind tunnel inclination. In order for an experiment to be considered valid the red isotope depletion slope should be steeper than the pre- and post-flight slopes in green. Note how the CO_2 production is lower in the post- than in the pre-flight period. Thus, the post-flight period was used for calculating flight power and recovery power (see Materials and Methods in the main manuscript).

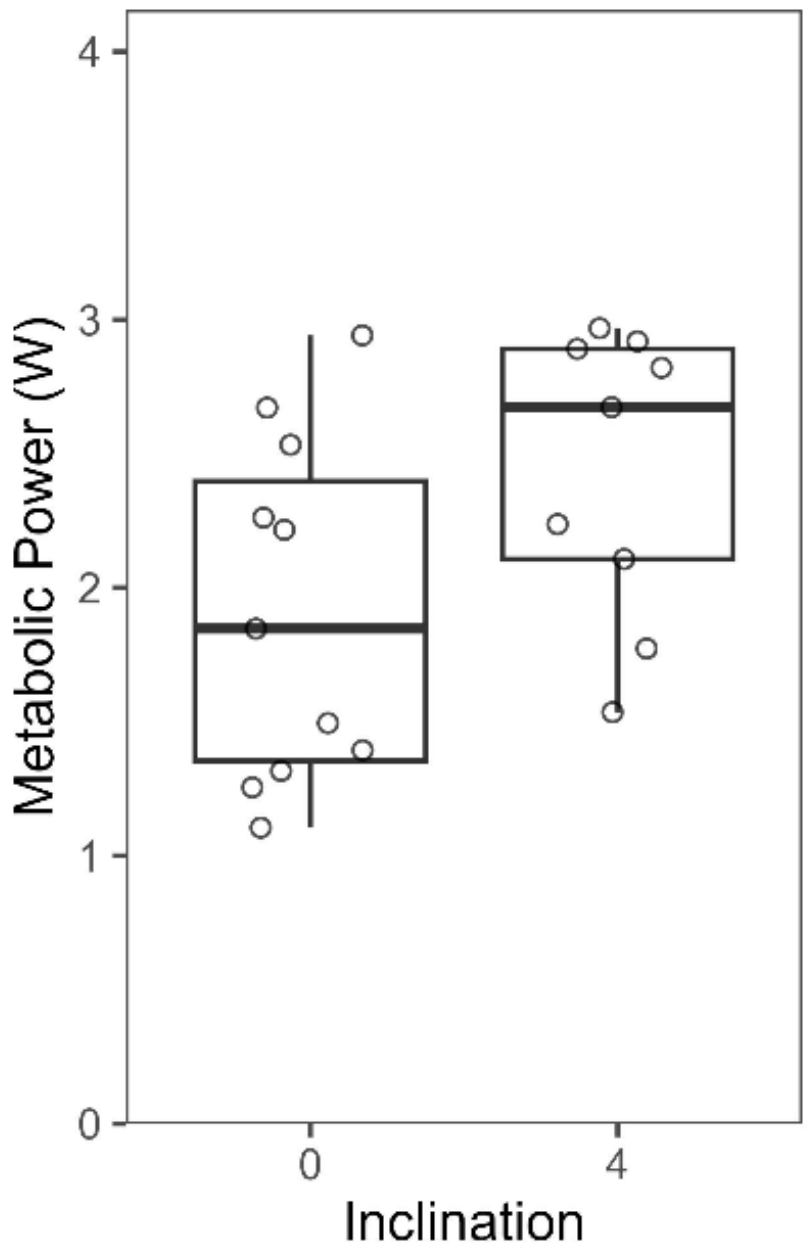

Fig. S2. Flight metabolic power in barn swallows flying in a wind tunnel at horizontal flight (0 degrees) and at a positive climbing flight (4 degrees). Each point represents a metabolic power measurement during one flight experiment. Only one flight speed was used for comparing horizontal and climbing flights: 11.4 m s^{-1}.

Table S1. Individual-level differences in metabolic power between horizontal and inclined flight (4 degrees positive). Values are mean flight metabolic power estimated from the NaBi method for each bird at horizontal flight and inclined flight at. Only one flight speed was used: 11.4 m s^{-1}.

Bird ID	n horizontal	n inclined (4 degrees)	Mean flight metabolic power horizontal (W)	Mean flight metabolic power inclined (W)	Δ flight metabolic power (W)
**62**	1	2	2.67	2.30	-0.375
**63**	3	1	1.27	2.67	1.40
**65**	2	1	2.58	2.97	0.389
**67**	1	2	1.85	2.21	0.366
**69**	1	2	1.49	2.58	1.08
**70**	3	1	2.02	2.11	0.090

Journal of Experimental Biology • Supplementary information

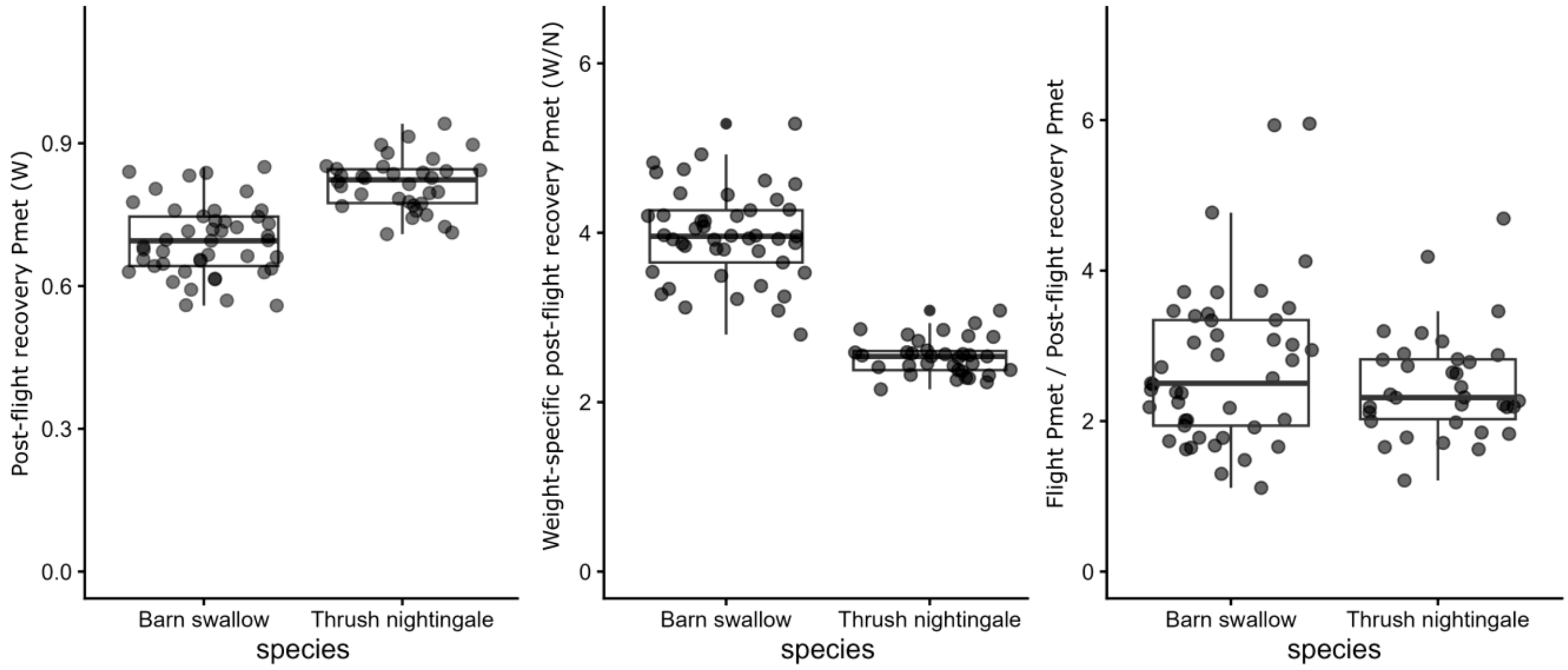

Fig. S3. Boxplots comparing post-flight (recovery) metabolic power (*P_{rec}*; left) weight-specific post-flight metabolic power (*P^*_{rec}*; middle) and the ratio between flight metabolic power and post-flight metabolic power and between barn swallows and thrush nightingales (flight *P_{met}* / *P_{rec}*; right). The post-flight (recovery) metabolic power is measured right after the flight and likely represents each species metabolic rate at background.

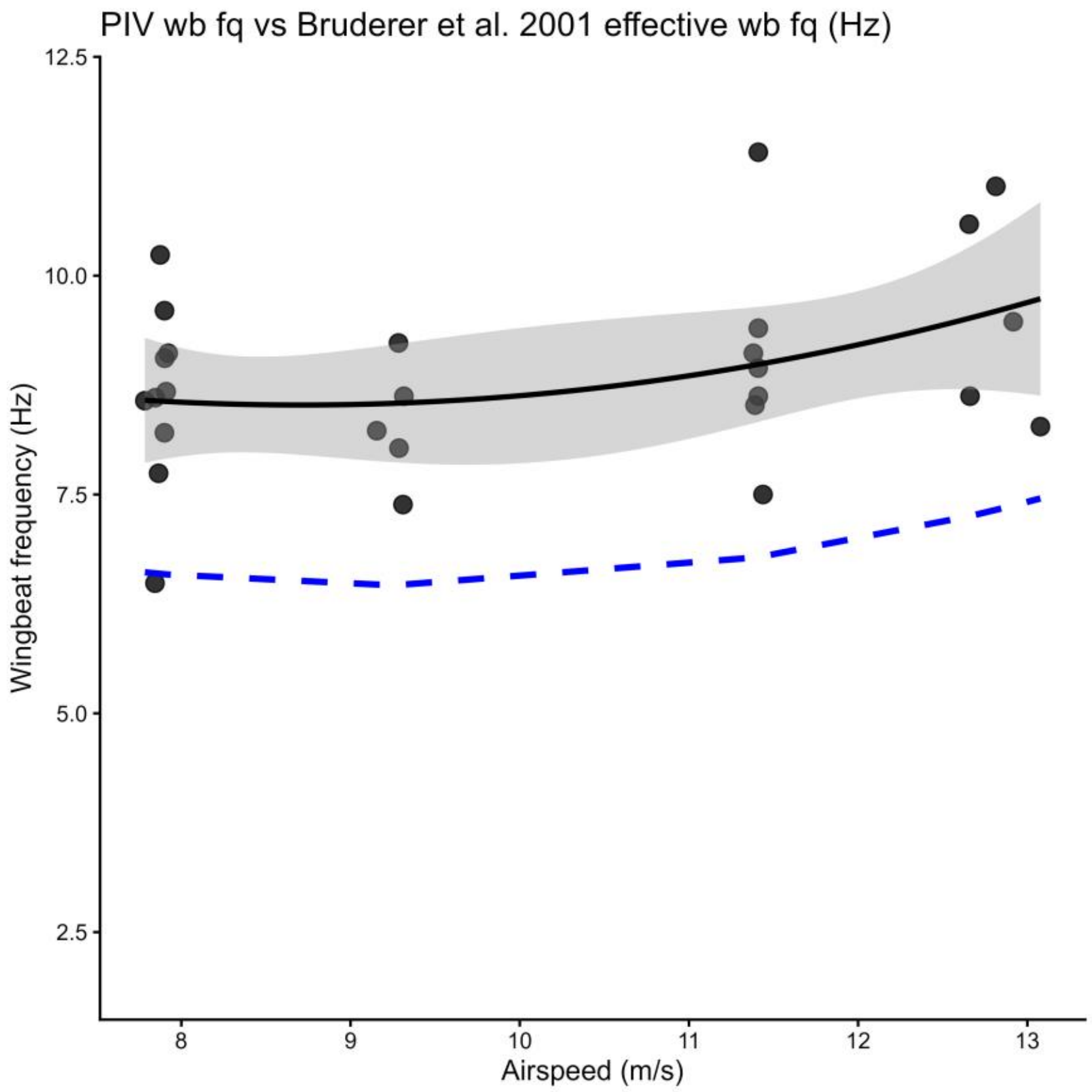

Fig. S4. Comparison between wingbeat frequency measured during active PIV flapping sequences in this study (black) and effective wingbeat frequency estimated from Bruderer et al. (2001) for barn swallows in horizontal flight (blue). Points show observed wingbeat frequencies from the present study. The shaded area representing the 95% confidence interval. The blue dashed line shows the estimated effective wingbeat frequency, F_{eff}, from Bruderer et al. (2001), evaluated across the same airspeed range. Observed wingbeat frequencies during active PIV sequences were higher than Bruderer's effective wingbeat frequencies, consistent with mainly using sequences without intermittent pauses between wingbeats in the PIV analysis.

Journal of Experimental Biology • Supplementary information

Supplementary Materials and Methods

Model selection for flight metabolic and mechanical power

Linear and polynomial mixed effect models including bird id as a random effect resulted in a singular fit. Therefore, we proceeded with the fixed-effects models only. A quadratic model fit significantly better than a linear model for raw metabolic power (likelihood-ratio test, $\Delta df = 1$, Δ Residual Sum of Squares = 3.094, $p = 0.012$). Adding the quadratic term significantly improves fit over a linear model for the weight-specific metabolic power (likelihood-ratio test: $\Delta df = 1$, $\Delta RSS = 108.19$, $p = 0.018$). Mechanical power increased nonlinearly with speed: a quadratic model fit significantly better than a linear model (likelihood ratio test, $\Delta df = 1$, $\chi^2 = 0.0093$, $p = 0.021$). Lift-specific mechanical power varied nonlinearly with airspeed; a quadratic fit outperformed a linear model (likelihood ratio test, $\Delta df = 1$, $\Delta RSS = 0.327$, $p = 0.0018$).

References

Bruderer, L., Liechti, F. and Bilo, D. (2001). Flexibility in flight behaviour of barn swallows (*Hirundo rustica*) and house martins (*Delichon urbica*) tested in a wind tunnel. J. Exp. Biol. 204, 1473-1484. doi:10.1242/jeb.204.8.1473

Journal of Experimental Biology • Supplementary information
